# Statistical tests for natural selection on regulatory regions based on the strength of transcription factor binding sites

**DOI:** 10.1186/1471-2148-9-286

**Published:** 2009-12-09

**Authors:** Alan M Moses

**Affiliations:** 1 Departments of Cell & Systems Biology and Ecology & Evolutionary Biology, University of Toronto, 25 Willcocks Street, Toronto, ON M5S 3B2, Canada

## Abstract

**Background:**

Although *cis*-regulatory changes play an important role in evolution, it remains difficult to establish the contribution of natural selection to regulatory differences between species. For protein coding regions, powerful tests of natural selection have been developed based on comparisons of synonymous and non-synonymous substitutions, and analogous tests for regulatory regions would be of great utility.

**Results:**

Here, tests for natural selection on regulatory regions are proposed based on nucleotide substitutions that occur in characterized transcription factor binding sites (an important type functional element within regulatory regions). In the absence of selection, these substitutions will tend to reduce the strength of existing binding sites. On the other hand, purifying selection will act to preserve the binding sites in regulatory regions, while positive selection can act to create or destroy binding sites, as well as change their strength. Using standard models of binding site strength and molecular evolution in the absence of selection, this intuition can be used to develop statistical tests for natural selection. Application of these tests to two well-characterized regulatory regions in *Drosophila *provides evidence for purifying selection.

**Conclusion:**

This demonstrates that it is possible to develop tests for selection on regulatory regions based on the specific functional constrains on these sequences.

## Background

The importance of *cis*-regulatory regions in the evolution of complex organisms is increasingly appreciated (reviewed in [1] and [2]), and general understanding of the molecular evolution of these sequences has grown rapidly [3-13]. An important outstanding question is whether natural selection has driven evolutionary changes in cis-regulatory regions, or whether these result from non-adaptive processes [14].

Many tests for natural selection can be applied to non-coding DNA and several important studies have identified signatures of natural selection in well-characterized regulatory regions (reviewed in [15]). Tests for selection on differences between species often compare the ratio of substitutions in transcription factor binding sites (an important class of functional element within cis-regulatory regions) to the surrounding non-coding DNA [16]. These tests are modelled after tests on coding regions that compare the patterns of amino acid changing differences to synonymous differences, which are amongst the most widely used and most powerful tests to detect the effects of natural selection on individual protein coding genes [17]. However, in applying these tests to binding sites, several important caveats must be considered [15]. In particular, it must be assumed that all of the functional elements in a regulatory region have been characterized, and that these remain constant in all species considered.

Here I develop a new approach to detect selection on individual *cis*-regulatory regions that takes advantage of the specificity of transcription factors to assign functional impact to nucleotide changes in binding sites. Recently, evolutionary analyses of large sets of transcription factor binding sites have highlighted the importance of considering the binding affinity or strength of the binding sites for their appropriate transcription factor [10,11,13,18]. Specifically, sequence differences in transcription factor binding sites can increase protein-DNA affinity, decrease it, or have no effect. In the absence of selection, fixation of random mutations will tend to decrease the strength of binding sties [19,20], whereas purifying selection will tend to preserve binding sites, such that the effects of subsequent fixations will cancel out [18]. On the other hand, though binding sites can arise in regulatory sequences as a result of the action of positive selection [19-21] or through genetic drift alone [22], I show that an increase in binding affinity on average is not expected in the absence of selection. I therefore propose to use the distribution of changes in strength of transcription factor binding sites to develop tests for natural selection on regulatory regions where the binding sites have been identified.

I analyze the fixed differences in two well-characterized regulatory regions in *Drosophila *(the *hb *anterior activator and the *eve *stripe 2 enhancer). These tests reveal statistical evidence for conservation of *cis*-regulatory information, which is consistent with the known conservation of function of these regulatory sequences.

## Results

### Quantifying the effects of substitutions in regulatory regions

Motivated by the power of tests for natural selection that exploit the constraints imposed on coding sequences by the genetic code, I sought to develop a test for natural selection on regulatory regions that takes into account the specific constraints on these regions: binding by transcription factors. Using standard matrix models for DNA binding specificity (known as Position Weight Matrices or Position Specific Scoring Matrices [23]), the binding energy of the interaction between a transcription factor and DNA is given by a sum of independent contributions from each residue at each position [23]. An estimate of the relative affinity or 'strength' a transcription factor binding site *X *of length *w *for its binding protein can be quantified using

Where *X*_*ib *_= 1 if the sequence *X *is nucleotide *b *at position *i *and 0 otherwise, *f*_*ib *_is the probability of observing nucleotide *b *at position *i *in a binding site for a transcription factor (from the specificity matrix), and *g*_*b *_is the probability of observing nucleotide *b *in the genomic background distribution [23].

Alternatively, the strength of the transcription factor binding sites in a region can be considered the regulatory information in that region, and the formula above can be motivated by information theoretic arguments [23]. Note that the framework and tests for selection presented here can equally be applied to information contained in the *cis*-regulatory region as to binding affinity. However, because recent work has focused on binding affinity (e.g., [11,13]) this work is presented from that perspective.

In order to quantify the effects of evolutionary changes in binding sites, I consider the effect of a single nucleotide change. In this case I define

associated with a change from base *a *to base *b *(*a*, *b *in {*A*, *C*, *G*, *T*}) where, once again, *i *is the position in the motif, *g *are background probabilities, and *f *are the probabilities in the specificity matrix model. Extending these methods to the general case of arbitrary numbers of substitutions is an area for further research (see Discussion).

### The effect of substitutions on binding sites in the absence of selection

**Figure 1:**
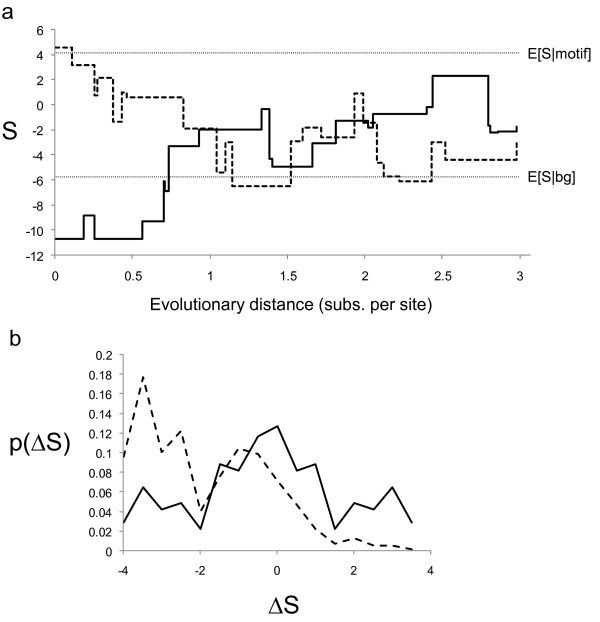
**Changes in binding site strength in the absence of selection**. a) shows time courses for the strength of a transcription factor binding site (S) in a simulation of evolution without selection. The strength of a real binding site (dotted trace) usually decreases from the strength expected for real binding sites (E[S|motif]) to that expected under background residue frequencies (E[S|bg]). An example of a binding site created from background sequence in the absence of selection (solid trace) is also shown. b) shows the probability (p) of observing a change in score of size ΔS given than a sequence is a binding site (dotted trace) or a background sequence (solid trace).

Most random mutations will decrease the strength of a transcription factor binding site, and therefore substitutions in the absence of selection will tend to decrease the affinity [19,20]. This follows from the fact that high affinity binding sites represent a small fraction of the possible sequences of a particular length. Since a substitution process that operates independently at each position in the sequence will tend to explore the majority sequence space, sequences that currently represent binding sites are much more likely to move away from these regions of sequence space than to remain in the relatively small regions of sequence space that represent binding sites. This implies that on average ΔS should be negative in the absence of selection. To illustrate this, I simulated the evolution of a binding site for Bcd (a developmental transcription factor in *Drosophila *whose specificity is well-characterized) under an HKY model (dotted trace in Figure 1a). The strength (S) of the binding site begins high (near the expected value of S for binding sites) and decays as substitutions eventually hit the critical residues. Consistent with this, the distribution of the changes in score (ΔS) is concentrated on values less than zero (dotted trace in Figure 1b).

### The effect of substitutions in binding sites under selection

In contrast, in functionally constrained regulatory regions, purifying selection will preferentially remove nucleotide changes that greatly alter the affinity of the binding sites [6,13]. When these substitutions do become fixed (albeit rarely), positive selection will tend to fix additional nucleotide changes that restore the binding affinity [18]. This process will tend to preserve the binding affinity, and ΔS will therefore tend to be zero if the regulatory region is under functional constraint.

Finally, consider adaptive evolution, which could have arbitrary effects on ΔS. For example, new transcription factor binding sites could be created from background sequence through successive adaptive fixations that increase binding site strength; this would lead to an increase in S, and therefore ΔS would be greater than zero on average. However, because new binding sites can also appear by genetic drift [21,22] it is possible that ΔS can be greater than zero in the absence of selection. To illustrate this, I simulated a background sequence of length equal to the Bcd binding site under the same HKY model as above, and found examples where binding sites arose in the absence of selection (Figure 1a solid trace). I argue that, although arbitrarily strong binding sites (high values of S) can be generated in the absence of selection, the distribution of changes in score (ΔS) is specified by the substitution process. Interestingly, since evolution in the absence of selection is unbiased with respect to the strength of the binding site, the distribution of changes in score is symmetric, with mean equal to zero (Figure 1b solid trace). This indicates that in the absence of selection, in background sequences we expect changes in score to cancel out. Therefore, while the creation of binding sites from background sequence cannot be considered evidence for positive selection, if the distribution of ΔS observed is statistically different from the pattern expected in the absence of natural selection, this can only be consistent with adaptive evolution.

Creation of new binding sites in regulatory regions is an intuitive case of adaptive regulatory evolution. However, depending on the situation, natural selection could also favour mutations that remove functional binding sites within a regulatory region, thus leading to an average ΔS of less than zero. Therefore, although a decrease in S on average is expected in the absence of selection, it could also occur in the presence of selection. Nevertheless, if ΔS is more negative than expected in the absence of selection, we have evidence that natural selection must be acting to remove binding sites.

In summary, for substitutions in a set of characterized binding sites we expect:

ΔS < 0 in the absence of constraint or adaptive destruction of binding sites

ΔS = 0 in the presence of functional constraint

ΔS > 0 during the creation of new binding sites (due to selection or genetic drift)

### Statistical tests for natural selection in regulatory regions

An attractive feature of using ΔS for a single substitution (as defined above) in a test for natural selection on regulatory regions is that its distribution can be computed exactly under standard models of molecular evolution in the absence of selection (see Methods, Figure 1b). I therefore propose to use the distribution of ΔS to test for the presence of natural selection on regulatory regions. If the distribution of ΔS is significantly different from that expected in the absence of selection, we can rule out the null hypothesis of evolution in the absence of selection.

Here I consider the tests for selection in the following cases.

1. If the observed ΔS in real binding sites is greater on average than ΔS expected for binding sites in the absence of selection, this indicates purifying selection to retain binding sites.

2. If the observed ΔS in real binding sites is less on average than ΔS expected for binding sites in the absence of selection, this indicates adaptive destruction of binding sites.

3. If the observed ΔS in real binding sites is greater on average than the ΔS expected for binding sites arising from background sequence in the absence of selection, this indicates adaptive creation of new sites.

Case 1: Here the pattern of evolution is consistent with purifying selection to preserve the function of the binding sites in the regulatory region. To rule out the null hypothesis of no constraint, we must compare the observed values of ΔS to the distribution of ΔS in sequences we know to be transcription factor binding sites, but in the absence of selection.

In the case of binding sites evolving in the absence of constraint:

where *E*[*X*] and *V*[*X*] are the mean and variance of the random variable *X*, respectively,

and *P*_*ab *_is the probability of substitution between bases *a *and *b *(i.e., *a*, *b *in {*A*, *C*, *G*, *T*}), computed under a standard model of molecular evolution, such that *P *= *e*^*Rt *^where *R *are the instantaneous rates of substitution and *t *is time (see Methods). The dotted trace in Figure 1b shows the distribution of ΔS for binding sites evolving in the absence of constraint.

In a practical setting, we expect to have observed some relatively modest number (*N*) of substitutions in characterized binding sites. Therefore, in order to test the significance of a set of observed ΔS values, I propose the statistic:

where *k *indexes *N *observed values of ΔS.

Since we can compute the mean and variance of ΔS under standard models of evolution (see Methods), according to the central limit theorem this statistic should be normally distributed with mean = 0 and variance = 1(the standard normal) under the hypothesis that the model of evolution is correct. We can therefore perform a one-tailed test that the observed mean is greater than that expected in the absence of selection.

**Figure 2:**
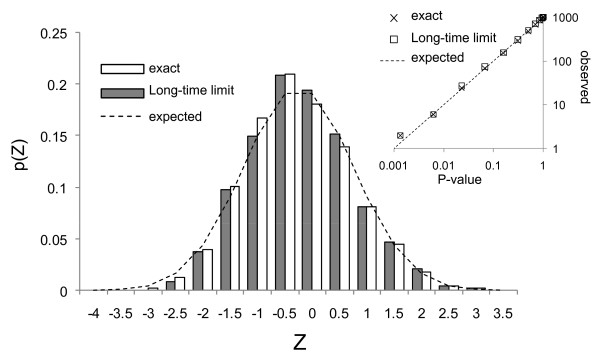
**Distribution of the proposed statistic under the null hypothesis**. In a simulation of molecular evolution under the null hypothesis (see text for details) the statistic proposed shows good agreement with the expected standard normal behavior (dotted trace) either using the mean and variance of ΔS computed exactly (unfilled bars) or in the long-time limit (filled bars). Inset is a comparison of the P-value as computed under the standard normal assumption and the number of times that value of statistic or greater was observed in 1000 simulations, using either the exact (Xs) or long-time limiting (squares) values for the mean and variance of ΔS.

I sought to confirm that the distribution of this statistic was as expected, particularly in the case of small *N *(few observed substitutions in binding sites) which is typical of real datasets. To simulate the null hypothesis of binding sites evolving in the absence of constraint, I simulated molecular evolution of the 6 real Bcd sites in the *hb *anterior activator under an HKY model with the transition-transversion rate ratio estimated from the alignment of the *hb *anterior activator (see methods) and evolutionary distance scaled so that we would observe approximately 5 substitutions in the 6 binding sites. I computed Z using E[ΔS] and V[ΔS] either under this model, and I observed good agreement with the expected standard normal behavior (Figure 2, 'exact').

Case 2: If we wish to test for adaptive destruction of transcription factor binding sites in a regulatory region, the average of ΔS should be significantly less than expected in the absence of selection. To test for this, we can perform a one tailed test using the statistic defined above, but in the opposite direction.

Case 3: If the average ΔS in a regulatory region is greater than 0, we wish to test whether the average ΔS is greater than we would expect to observe in the absence of selection. Now the null hypothesis is that the average increase in binding affinity we have observed is due binding sites arising in background sequence by genetic drift. Once again the distribution of ΔS can be computed exactly, and the mean and variance are:

with

The solid trace in Figure 1b shows this distribution. This distribution is symmetric, and the expectation is zero. This follows from the fact that the substitution processes in the absence of selection is unbiased with respect to the binding site strength, and that the residue frequencies in background genomic sequence are assumed to be drawn from the equilibrium of the substitution process. The means and variances can be used to form a Z-statistic as illustrated above, and simulations again confirm the expected distribution of the statistic (data not shown). If the observed average ΔS is significantly greater than expected in the absence of selection, we find evidence for adaptive evolution. For example, for the 20 substitutions shown in Figure 1a (solid trace) the average ΔS is 0.45, which gives Z = 0.97 and is not significant. Thus, although there is a large change in S, the pattern of changes is consistent with the absence of selection.

### An approximation to the distribution of ΔS

Under substitution models with no transition-transversion bias [24], the distribution of ΔS does not depend on evolutionary distance. For example, I can show (see Methods) that for binding sites evolving in the absence of selection,

A similar, albeit more complicated expression is available for the variance (see Methods). These expressions depend only on the equilibrium probabilities of the four nucleotides and the probabilities in the specificity matrix model for the transcription factor.

**Figure 3:**
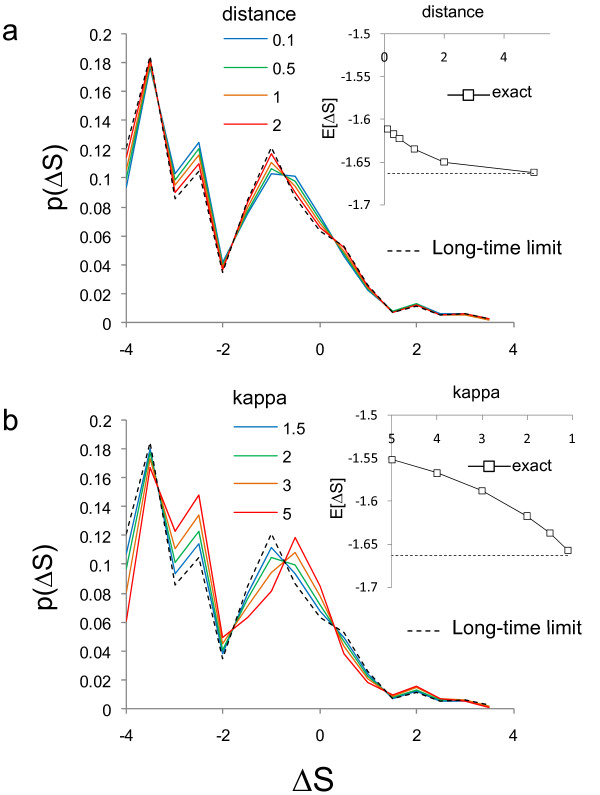
**Dependence of the distribution of ΔS on evolutionary parameters**. a) shows the probability distribution of ΔS as evolutionary distance varies (coloured solid traces) for the Bcd matrix under an HKY model with transition-transversion rate ratio set to 2. The distribution rapidly converges to the long-time limit distribution (dotted trace). b) shows the probability distribution of ΔS as the transition-transversion rate ratio (kappa) varies (coloured solid traces) for the Bcd matrix under an HKY model with evolutionary distance set equal to 0.3 substitutions per site. Once again, the distribution converges to the long-time limit (dotted trace). Inset in both is the convergence of the mean of ΔS (squares) to the long-time limit (dotted trace). Distributions are for real binding sites evolving in the absence of selection.

In the general case, the distribution of ΔS depends very weakly on the evolutionary distance (Figure 3a) and only somewhat more strongly on the transition-transversion bias (Figure 3b). It is therefore possible to obtain a good approximation of the distribution of ΔS using the formulas obtained under the simpler substitution models. I refer to this approximation of the distribution of ΔS as the 'long-time limit' because it becomes exact in the limit of long evolutionary time even in the presence of transition-transversion bias (Figure 3). As expected, using the long-time limit E[ΔS] and V[ΔS] when calculating the Z statistic described above also gives the standard normal behaviour (Figure 2, 'Long time limit'). Thus, this approximation allows application of tests based on the distribution of ΔS without estimates or assumptions about the evolutionary process in the absence of selection.

### Application to the hb anterior activator

**Figure 4:**
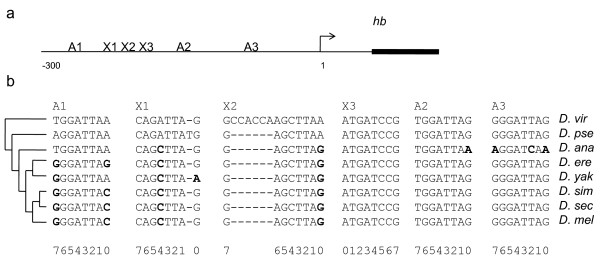
**Characterized Bcd sites in the *hb *anterior activator**. a) schematic of the Bcd sites in the *hb *anterior activator. b) alignments of the binding sites. Bold residues indicate the substitutions that were included in the analysis. Numbers below the alignments indicate the relative positions in the Bcd binding motif. Abbreviations are *D. - Drosophila, vir - virilis, pse - pseudoobscura, ana - ananassae, ere - erecta, yak - yakuba, sim - simulans, sec - sechelia, mel - melanogaster*

The *hb *anterior activator (Figure 4a) responds to the Bcd gradient in the early *D. melanogaster *embryo [25]. It is thought to have been conserved since the divergence of *D. melanogaster *and *D. virilis *[26] and contains well-defined binding sites for Bcd [27]. We therefore expect to see evidence of functional constraint on this regulatory region.

Using *D. virilis *and *D. pseudoobscura *as outgroups, I identified 10 substitutions in the alignment of the 6 well-characterized Bcd binding sites (Figure 4b) and used equation 2 above to compute ΔS for each substitution (Table 1). The average ΔS for these substitutions was -0.31(s.d. = 1.07). To test for functional constraint (case 1), I used equations 3-5 above to compute E[ΔS] = -1.61 and V[ΔS] = 2.77 for the Bcd matrix, using as the null hypothesis an HKY model with parameters (kappa = 2.26 and total evolutionary distance = 0.36 subs) estimated from an alignment of the entire regulatory region (see Methods). The test above yields Z = 2.48, which is significant with P-value < 0.01 (Table 2). As expected, similar results are obtained using the long-time limit distribution of ΔS (Table 2).

**Table 1:** Substitutions in Bcd sites in the *hb *anterior activator

From	To	Pos	ΔS	site	lineage	coordinate
T	G	7	1.58	A1	*mel *Subgroup	3R:4520596

A	G	0	0.29	A1	*ere*	3R:4520603

A	C	0	-0.88	A1	*sim*/*sec*/*mel*	3R:4520603

G	A	0	-0.29	X1	*yak*	3R:4520561

A	C	4	-0.22	X1	*mel *Group	3R:4520557

A	G	0	0.29	X2	*mel *Group	3R:4520545

G	A	0	-0.29	A2	*ana*	3R:4520493

G	A	0	-0.29	A3	*ana*	3R:4520388

T	C	2	-2.69	A3	*ana*	3R:4520386

G	A	7	-0.57	A3	*ana*	3R:4520381

Positions in the Bcd binding motif (Pos) are numbered as shown in Figure 4. Abbreviations for lineages are as in Figure 4. Coordinates are based on mapping of sites to the *D. melanoaster *genome [52]

**Table 2:** Tests for selection on the *hb *anterior activator

*N*	E[ΔS]	V[ΔS]	Observed average ΔS	Case	Z	P-value	Notes
10	-1.61	2.77	-0.31	1	2.48	0.007	Phylogenetic model estimated from *hb *anterior activator alignment

10	-1.66	2.87	-0.31	1	2.53	0.005	Long time limit distribution of ΔS

7	-1.61	2.77	0.07	1	2.67	0.004	Excluding A3, phylogenetic model estimated from *hb *anterior activator alignment

5	-1.61	2.77	0.20	1	2.43	0.007	Excluding A3 and substitutions on the lineage leading to the melanogaster group, phylogenetic model estimated from *hb *anterior activator alignment

5	0	4.26	0.20	2	0.22	0.415	As above, test for adaptive evolution

I noted that 3 substitutions had occurred in a single binding site on a single lineage (A3, Table 1) and was concerned that this might indicate that the assumption of single substitutions at each site was invalid on this lineage. I therefore performed the test excluding these substitutions and found similar results (Table 2). I also found evidence for constraint when excluding substitutions along the relatively long branch leading to the *melanogaster *group. Noting that removing the substitutions led to an average ΔS for the region greater than 0, I tested for evidence for adaptive creation of binding sites in this regulatory region (case 3). However, performing the test above (equations 7-9) yielded Z = 0.22, which is not significant, indicating that the observed increase in binding strength could have been observed in the absence of selection.

### More complex regulatory regions

The *hb *anterior activator serves as a good test case for this method because it contains multiple binding sites for the same transcription factor. However, in general regulatory regions contain multiple binding sites for multiple different transcription factors. Note that the arguments above regarding the expected ΔS in regulatory regions apply regardless of whether the binding sites are for a single transcription factor or many different transcription factors.

To extend the statistical test to regulatory regions with multiple binding sites for different factors, two approaches are possible. If enough substitutions in each type of binding site are present, the test above can be performed for each type, and then their results can be combined. However, in the case of few substitutions, it may be preferable to pool the substitutions first. To do so, we must compute the distribution of ΔS expected from a mixture of transcription factor binding sites. ΔS is now drawn from a *v *component mixture model,

where *v *is the number of types of transcription factor binding sites, *π*_*i *_is the probability that the substitution occurred in the *j*-th type, and *p*(ΔS)_*j *_is the distribution of ΔS for the *j*-th type of binding motif. We can compute these *π*_*j *_for any regulatory region given the numbers of each type of binding site in a characterized regulatory region (see Methods):

where *n*_*j*_, *f*_*j *_and *w*_*j *_are the number of occurrences, specificity matrix and width of the *j*-th type of binding site, and *P *is a substitution matrix as above. Since it is possible to compute the means and variances of mixture distributions as a func tion of the component distributions (see Methods),

we can apply the test suggested above in the mixture case as well. Once again, we can obtain a good approximation to this distribution using the long-time limit. To confirm the distribution of the test statistic proposed above in the case of mixtures of binding sites, I again performed simulations under the null hypothesis of no constraint, this time using the 6 Kr sites and 5 Bcd sites in the *eve *stripe 2 enhancer, and once again found the expected standard normal behavior (data not shown).

### Application to the eve stripe 2 enhancer

The individual binding sites in the *eve *stripe 2 enhancer are well-characterized [28,29] and this enhancer exemplifies complex *cis*-regulatory sequences in that it contains multiple binding sites for multiple transcription factors [30]. Here I consider the 5 Bcd binding sites and 6 Kr binding sites to illustrate the application of the test for selection to a complex regulatory region (see Additional file 1 for alignments of these binding sites). Two of the Kr and Bcd binding sites the eve stripe 2 enhancer overlap, and I excluded two substitutions that affect the strength of more than one binding site (Table 3).

**Table 3:** Substitutions in Bcd and Kr sites in the *eve *stripe 2 enhancer

From	To	Pos	ΔS	site	lineage	Coordinate
G	C	N.A.	N.A.	BC-5, KR-5	*ana*	2R:5489670

G	T	0	0.62	KR-5	*ana*	2R:5489674

A	G	0	-0.44	KR-4	*mel*	2R:5489850

C	G	3	-0.51	KR-4	*ana*	2R:5489853

G	C	N.A.	N.A.	KR-3, BC-1	*ere*/*yak*	2R:5490048

C	T	3	-0.92	KR-2	*mel *Subgroup	2R:5490098

A	G	0	-0.44	KR-2	*ana*	2R:5490094

T	C	9	-1.39	KR-1	*ana*	2R:5490140

A	G	7	-2.73	KR-1	*ana*	2R:5490142

A	G	0	0.12	BC-3	*yak*	2R:5489835

A	C	0	1.22	BC-3	*sim*/*sec*	2R:5489835

T	C	1	-1.67	BC-3	*yak*	2R:5489836

A	G	2	-2.30	BC-3	*ere*	2R:5489837

T	C	3	0.41	BC-3	*yak*	2R:5489838

T	A	3	3.85	BC-3	*mel*/*sim*/*sec*	2R:5489838

C	G	7	0.48	BC-3	*yak*	2R:5489842

C	G	7	-0.48	BC-4	*mel*/*sim*/*sec*	2R:5489683

G	C	7	0.48	BC-4	*sim*	2R:5489683

N.A. - not applicable, other abbreviations are as in Figure 3. Naming of binding sites is as in [31]. Coordinates are based on mapping of sites to the *D. melanoaster *genome [52].

This left 16 substitutions (9 in bcd binding sites and 7 in Kr binding sites) for which I used equation 2 to compute associated ΔS values (Table 3). The average ΔS for all the substitutions was -0.23, and I performed the test described above with evolutionary distance and transition-transversion rate ratio estimated from the alignment of the *eve *stripe 2 enhancer (see methods). Using equations 11-14, I computed the distribution of ΔS for 6 Kr sites and 5 Bcd sites evolving in the absence of constraint. This yields E[ΔS] = -1.56 and V[ΔS] = 2.53, and provides evidence for constraint (case 1) on the regulatory sequence with Z = 2.53 and P-value = 0.0004 (Table 4).

**Table 4:** tests for selection on the *eve *stripe 2 enhancer

*N*	E[ΔS]	V[ΔS]	Observed average ΔS	Case	Z	P-value	Notes
16	-1.56	2.53	-0.23	1	3.35	0.0004	Phylogenetic model estimated from *eve *stripe 2 alignment

16	-1.60	2.59	-0.23	1	3.41	0.0003	Long time limit distribution of ΔS

9	-1.56	2.53	0.05	1	3.04	0.001	Phylogenetic model estimated from *eve *stripe 2 alignment, excluding substitutions on lineages with multiple substitutions in individual binding sites

6	-1.56	2.51	-0.39	1	1.81	0.035	Phylogenetic model estimated from *eve *stripe 2 alignment, excluding BC-3 and substitutions on lineages with multiple substitutions in individual binding sites

Although its function has been conserved over evolution [31], the *eve *stripe 2 enhancer has undergone some linage specific evolution [32], as well as gained and lost individual binding sites; its evolution is characterized by rapid sequence divergence [31,33]. Consistent with this, the alignments of *D. pseudoobscura *for BC-3 were not possible, as this site seems to have appeared recently [32]. Within the closely related species in the melanogaster subgroup, BC-3 contains seven inferred substitutions, four of which are inferred to occur along the lineage leading to *D. yakuba*. In addition to the rapid divergence of BC-3, I again found cases where more than one substitution had occurred along the *D. ananassae *lineage in a single binding site. In addition, I therefore performed the tests excluding lineages with multiple substitutions, or excluding BC-3 entirely. In all cases there is still sufficient power to provide statistical evidence against the null hypothesis of no constraint (table 4). In no case could I find evidence for adaptive evolution (case 2 or case 3, data not shown).

## Discussion and Conclusion

### A new test for natural selection on regulatory regions

One of the difficulties in many current evolutionary analyses of *cis*-regulatory regions is that it is difficult to choose an appropriate set of unconstrained sites to which to compare the functional regulatory sites. In general, studies either choose the rate of substitution in surrounding non-coding sequence [16] or in synonymous sites in adjacent protein coding regions [34]. Both assumptions may be problematic. The former assumes that the surrounding DNA is under no functional constraint (as opposed to some unknown constraints). In the latter case, because non-coding sequences show larger numbers of insertions and deletions than coding regions, it is not always clear that rate estimates based on alignments of coding and non-coding regions can be directly compared.

Tests based on the distribution of ΔS, such as those proposed here, do not rely on these assumptions, as they consider only substitutions that occur in binding sites. Practically, this is an attractive feature of these tests, as they only require accurate alignments of the binding sites, which are generally more reliable than alignments of unconstrained non-coding DNA [35].

Another attractive feature of tests based on the distribution of ΔS in the absence of selection is that they make few assumptions about the nature of selection on binding sites. For example, it is not assumed that binding sites are all under the same strength of selection, or that they all have the same binding affinity - only the changes in strength of binding are important. Further, even under a stabilizing selection model, where binding sites for a given transcription factor are gained and lost over evolution [33], ΔS will be zero on average if the total output of the regulatory sequence is preserved: the negative ΔS associated with the binding site loss will be compensated by positive ΔS associated with the binding site gain. However, if binding sites for one transcription factor are replaced by binding sites for another, ΔS may no longer be zero on average and testing for selection in this case is an area for further research.

### Practical considerations, limitations and future improvements

Application of these tests to two well-characterized regions demonstrates that they have enough power to detect constraint on individual regulatory regions with ~ 10 substitutions in binding sites, and perhaps even as few as 5 or 6 substitutions (tables 2 and 4). However, application to the *eve *stripe 2 enhancer illustrates several practical difficulties: First, I didn't include the Hb binding sites in this enhancer [36] because these binding sites contain homopolymeric runs, and it is difficult to assign a 'position' to a substitution; ΔS cannot be reliably computed for each substitution in this case. Second, although the *eve *stripe 2 enhancer has characterized sites for Gt, I did not include these because the sequence specificity of this transcription factor is poorly characterized. Third, the *eve *stripe 2 enhancer contains substitutions in overlapping binding sites, for which it is not clear how to calculate ΔS; these were therefore excluded from the analysis. Finally, the distribution of ΔS is sensitive to the estimation of the frequency parameters in the specificity matrix. For example, I excluded the Bcd binding sites in the *eve *stripe 2 enhancer and reconstructed the Bcd matrix for analysis of that regulatory region. If the binding sites in the regulatory region of interest are included in the estimation of the specificity matrix, there is a potential for circularity in the analysis. Thus, the tests require (i) well-characterized transcription factor binding specificity and (ii) confident alignment of a binding site to a single specificity matrix. None of these constraints are present for tests that compare binding sites to surrounding regions or synonymous sites [16,34] or for tests of natural selection based on spacing between conserved blocks [35-37]. However, rapid advances in methods to characterize DNA-protein interactions are making specificity information available for large numbers of transcription factors [38-40]. Among these are methods that yield information about binding to each sequence, such that the assumption of independent contributions to binding of each DNA base in the binding site could in principle be relaxed [38,41].

In addition, the tests I have proposed assume that only a single substitution has occurred at any position in binding sites. Although for most of the data analyzed here this assumption seems valid, I noted several cases were multiple substitutions occurred on a single lineage, suggesting the possibility of 'multiple hits' at a single site. Furthermore, there is clear evidence of insertions and deletions occurring near or within the binding sites considered here. These are likely to affect their binding affinity, but are not included in the null model of molecular evolution in the absence of selection. More sophisticated models of molecular evolution [42] may be able to account for these effects, and these could be applied in this framework. Similarly, the evolutionary models here do not account for di-nucleotide substitution bias, particularly the elevated rate of CpG to TpG found in mammals; these could be included using an improved null model [43,44].

Finally, I note that I have suggested one simple statistical test based on the observed average ΔS, however many tests based on distribution of ΔS are possible. For example, purifying selection might also be expected to reduce the variance of ΔS. Indeed, in the case of the Bcd sites in the *hb *anterior enhancer, the observed variance of ΔS is less than expected, though this difference is not significant (e.g., 1.15 vs. 2.86, n = 10, chi-square test *P *= 0.089). Determining what tests have the most power to detect various types of selection in regulatory regions is an area for further research. In general, however, it seems very likely that tests that consider the effects of substitutions on transcription factor binding site affinity will facilitate the detection of adaptive evolution in regulatory regions.

## Methods

### Construction of motif matrices

I used publically available compilations of characterized binding sites for Bcd and Kr [33,45] to construct specificity matrices using a pseudocount of 1 per position. Throughout this study, I use as the background distribution (g_A_, g_C_, g_G_, g_T_) = (0.3, 0.2, 0.2, 0.3) which is close to the observed nucleotide probabilities in *drosophila *non-coding DNA. In order to avoid the possibility of circularity, for analysis of each regulatory region I excluded the characterized sites from that region and reconstructed the matrix, such that (for example) Bcd sites from the *hb *anterior activator were not included in the matrix used for analysis of the *hb *anterior activator. These matrices were used to compute ΔS for each substitution (Tables 1 and 3) and E[ΔS] and V[ΔS] (Tables 2 and 4).

### Alignments and phylogenetic analysis of regulatory sequences

I obtained homologous regions for each regulatory region from the UCSC genome-browser alignments [46]. The sequences were then realigned using mLAGAN [47]. Using these alignments and the known species relationships for these species [48], I estimated the evolutionary distance and transition-transversion rate ratio bias under an HKY model [24] using paml [49]. The parameters estimated using paml for each regulatory region were then used to compute the exact E[ΔS] and V[ΔS] shown in Tables 2 and 4.

### Simulations of molecular evolution

To confirm that the test statistic had a standard normal distribution under the null hypothesis, I simulated the evolution of the 6 known binding sites in the *hb *anterior activator. To do so, I inferred the ancestral sequences using maximum parsimony [50], and then simulated their evolution by introducing substitutions using an HKY model with kappa estimated from the alignment, 60% AT content for the equilibrium distribution of nucleotides, and evolutionary distance scaled to observe an average of 5 substitutions over the 6 binding sites. I then computed the average ΔS for the substitutions we observed, and calculated the Z statistic using E[ΔS] and V[ΔS] computed exactly using the evolutionary model or using the long-time limit approximation. I repeated this simulation until I observed 1000 cases with at least 3 substitutions in total. Simulations for the *eve *stripe 2 enhancer were similar, except I used the actual *D. melanogaster *binding sites (because reliable inference of the ancestral sites was difficult) and that the evolutionary distance was scaled so that the 5 substitutions were distributed over the 5 Bcd and 6 Kr sites.

### Distribution of ΔS

I sought to compute the distribution of ΔS in the absence of selection. Because the number of observed evolutionary differences in any particular binding site is typically small, I make the assumption that each DNA difference in a transcription factor binding site occurs independently, and presence of a single change has no effect on the probability of other changes. Under this assumption, the probability of observing the particular change from base *a *to base *b *(*a*, *b *in {*A*, *C*, *G*, *T*}) at position *i *is

where *p*(*one subs*.) ≡ *φ*, and , so that

and *P *= *e*^*Rt *^is a substitution probability matrix. The expected value of ΔS for binding sites that evolve in the absence of selection is ΣΔ*S*_*iab *_*p*(*a *→ *b at i|one subs*.) or

Similarly, for the variance, we have

As computed here, the distribution of ΔS is exact only for the first substitution at each site in a particular sequence. Therefore it is important to apply the tests described here to cases where only small numbers of substitutions have occurred on each lineage. For the regulatory sequences considered here, this assumption seems appropriate. However, if enough substitutions have occurred such that multiple subsequent substitutions occur at the same position, the distribution of ΔS computed based on the sequence of a reference species or inferred ancestral sequence will no longer be exact. Computing the distribution of ΔS under more relaxed assumptions is area for further research.

Since I am considering the conditional probability that one particular substitution occurs out of all the possible substitutions that could have occurred, under some substitution models such as F81 [51], or in the limit of long evolutionary time, this probability does not depend on time and mutation rate (evolutionary distance). I refer to this time independent approximation as the 'long time limit' distribution, and derive formulas under this assumption. Under the F81 [51] substitution model *P*_*ab *_= *g*_*b*_(1 - *e*^-*ut*^), where *u *is the mutation rate and *t *is time. We have

and therefore *p*(*a *→ *b at i|one subs*.) = 

which depends only on the frequencies in the matrix, *f*, and the background distribution of nucleotides *g*, where now *a*, *b *and *c *index the bases {*A*, *C*, *G*, *T*}. Therefore under this model, the long time limit is exact. Substitution into the general formulas for the expectation gives

for the case of binding sites evolving in the absence of selection (case 1). This formula can be simplified using the fact that ΔS = 0 if *a *= *b*:

Therefore, we have for case 1,

To compute the variance, I use *V*[Δ*S*] = *E*[Δ*S*^2^] - *E*[Δ*S*]^2^, where

Similarly, for the case of background sequences evolving into binding sites in the absence of selection (the null hypothesis for case 3), the same calculations give E[ΔS] = 0, and

While these formulas are complicated, they depend only on the residue probabilities in the matrix (*f*) and the background (*g*), and therefore phylogenetic analysis is not required.

### Mixtures of binding sites

In the case of *v *transcription factors binding a regulatory region, ΔS is drawn from a mixture distribution,

where *v *is the number of types of transcription factor binding sites, *π*_*i *_is the probability that the substitution occurred in the *j*-th type, and *p*(ΔS)_*j *_is the distribution of ΔS for the *j*-th type of binding motif. To compute this we need *π*_*i *_= *p*(*subs. in type j *| *one subs*.), so

This can be computed using

where *w*_*i *_is the length of the *j*-th motif and *n*_*i *_is the number of times that motif occurs in the regulatory region. In this case

and therefore

for case 1. To compute the mean and variance of arbitrary mixture models we proceed as follows. To simplify the notation, I will indicate sums over *i*, *a*, *b*, as sums over ΔS. In this notation,

using the linearity of the expectation. For the variance,

We now add and subtract the square of E[ΔS] for the *j*-th motif.

We now reorder the terms and take the expectations out of the summations,

And finally

Scripts to compute E[ΔS] and V[ΔS] will be provided from the author's website.

## Supplementary Material

Additional file 1 ***eve *stripe 2 enhancer binding sites**. Alignments of binding sites in the *eve *stripe 2 enhancer Click here for file

## References

[B1] Prud'homme B Gompel N Carroll SB Emerging principles of regulatory evolution Proceedings of the National Academy of Sciences of the United States of America 2007 104 Suppl 1 8605 8612 10.1073/pnas.0700488104 17494759 PMC1876436

[B2] Wray GA The evolutionary significance of cis-regulatory mutations Nature Reviews Genetics 2007 8 3 206 216 10.1038/nrg2063 17304246

[B3] Wasserman WW Palumbo M Thompson W Fickett JW Lawrence CE Human-mouse genome comparisons to locate regulatory sites Nature Genetics 2000 26 2 225 228 10.1038/79965 11017083

[B4] Dermitzakis ET Clark AG Evolution of transcription factor binding sites in Mammalian gene regulatory regions: conservation and turnover Molecular Biology and Evolution 2002 19 7 1114 21 12082130 10.1093/oxfordjournals.molbev.a004169

[B5] Dermitzakis ET Bergman CM Clark AG Tracing the evolutionary history of Drosophila regulatory regions with models that identify transcription factor binding sites Molecular Biology and Evolution 2003 20 5 703 714 10.1093/molbev/msg077 12679540

[B6] Moses AM Chiang DY Kellis M Lander ES Eisen MB Position specific variation in the rate of evolution in transcription factor binding sites BMC Evolutionary Biology 2003 3 19 10.1186/1471-2148-3-19 12946282 PMC212491

[B7] Emberly E Rajewsky N Siggia ED Conservation of regulatory elements between two species of Drosophila BMC Bioinformatics 2003 4 57 10.1186/1471-2105-4-57 14629780 PMC302112

[B8] Sinha S Siggia ED Sequence turnover and tandem repeats in cis-regulatory modules in drosophila Molecular Biology and Evolution 2005 22 4 874 885 10.1093/molbev/msi090 15659554

[B9] Cameron RA Chow SH Berney K Chiu T Yuan Q Krämer A Helguero A Ransick A Yun M Davidson EH An evolutionary constraint: strongly disfavored class of change in DNA sequence during divergence of cis-regulatory modules Proceedings of the National Academy of Sciences of the United States of America 2005 102 33 11769 11774 10.1073/pnas.0505291102 16087870 PMC1188003

[B10] Mustonen V Lässig M Evolutionary population genetics of promoters: predicting binding sites and functional phylogenies Proceedings of the National Academy of Sciences of the United States of America 2005 102 44 15936 15941 10.1073/pnas.0505537102 16236723 PMC1276062

[B11] Mustonen V Kinney J Callan CG Lässig M Energy-dependent fitness: a quantitative model for the evolution of yeast transcription factor binding sites Proceedings of the National Academy of Sciences of the United States of America 2008 105 34 12376 12381 10.1073/pnas.0805909105 18723669 PMC2527919

[B12] Gaffney DJ Blekhman R Majewski J Selective constraints in experimentally defined primate regulatory regions PLoS Genetics 2008 4 8 e1000157 10.1371/journal.pgen.1000157 18704158 PMC2490716

[B13] Kim J He X Sinha S Evolution of regulatory sequences in 12 Drosophila species PLoS Genetics 2009 5 1 e1000330 10.1371/journal.pgen.1000330 19132088 PMC2607023

[B14] Lynch M The frailty of adaptive hypotheses for the origins of organismal complexity Proceedings of the National Academy of Sciences of the United States of America 2007 104 Suppl 1 8597 8604 10.1073/pnas.0702207104 17494740 PMC1876435

[B15] Hahn MW Detecting natural selection on cis-regulatory DNA Genetica 2007 129 1 7 18 10.1007/s10709-006-0029-y 16955334

[B16] Jenkins DL Ortori CA Brookfield JF A test for adaptive change in DNA sequences controlling transcription Proc Biol Sci 1995 261 1361 203 7 10.1098/rspb.1995.0137 7568273

[B17] Fay JC Wu C Sequence divergence, functional constraint, and selection in protein evolution Annual review of genomics and human genetics 2003 4 213 35 10.1146/annurev.genom.4.020303.162528 14527302

[B18] Mustonen V Lässig M From fitness landscapes to seascapes: non-equilibrium dynamics of selection and adaptation Trends in Genetics: TIG 2009 25 3 111 119 10.1016/j.tig.2009.01.002 19232770

[B19] Schneider TD Evolution of biological information Nucleic Acids Research 2000 28 14 2794 2799 10.1093/nar/28.14.2794 10908337 PMC102656

[B20] Berg J Willmann S Lässig M Adaptive evolution of transcription factor binding sites BMC Evolutionary Biology 2004 4 1 42 10.1186/1471-2148-4-42 15511291 PMC535555

[B21] MacArthur S Brookfield JF Y Expected Rates and Modes of Evolution of Enhancer Sequences Molecular Biology and Evolution 2004 21 6 1064 1073 10.1093/molbev/msh105 15014138

[B22] Stone JR Wray GA Rapid evolution of cis-regulatory sequences via local point mutations Molecular Biology and Evolution 2001 18 9 1764 1770 11504856 10.1093/oxfordjournals.molbev.a003964

[B23] Stormo GD DNA binding sites: representation and discovery Bioinformatics 2000 16 1 16 23 10.1093/bioinformatics/16.1.16 10812473

[B24] Yang Z Goldman N Friday A Comparison of models for nucleotide substitution used in maximum-likelihood phylogenetic estimation Molecular biology and evolution 1994 11 2 316 24 8170371 10.1093/oxfordjournals.molbev.a040112

[B25] Driever W Nüsslein-Volhard C The bicoid protein is a positive regulator of hunchback transcription in the early Drosophila embryo Nature 1989 337 6203 138 143 10.1038/337138a0 2911348

[B26] Lukowitz W Schröder C Glaser G Hülskamp M Tautz D Regulatory and coding regions of the segmentation gene hunchback are functionally conserved between Drosophila virilis and Drosophila melanogaster Mechanisms of Development 1994 45 2 105 115 10.1016/0925-4773(94)90024-8 8199047

[B27] Driever W Thoma G Nüsslein-Volhard C Determination of spatial domains of zygotic gene expression in the Drosophila embryo by the affinity of binding sites for the bicoid morphogen Nature 1989 340 6232 363 367 10.1038/340363a0 2502714

[B28] Small S Kraut R Hoey T Warrior R Levine M Transcriptional regulation of a pair-rule stripe in Drosophila Genes & Development 1991 5 5 827 839 10.1101/gad.5.5.827 2026328

[B29] Stanojevic D Small S Levine M Regulation of a segmentation stripe by overlapping activators and repressors in the Drosophila embryo Science 1991 254 5036 1385 1387 10.1126/science.1683715 1683715

[B30] Howard ML Davidson EH cis-Regulatory control circuits in development Developmental Biology 2004 271 1 109 118 10.1016/j.ydbio.2004.03.031 15196954

[B31] Ludwig MZ Patel NH Kreitman M Functional analysis of eve stripe 2 enhancer evolution in Drosophila: rules governing conservation and change Development 1998 125 5 949 958 9449677 10.1242/dev.125.5.949

[B32] Ludwig MZ Palsson A Alekseeva E Bergman CM Nathan J Kreitman M Functional evolution of a cis-regulatory module PLoS Biology 2005 3 4 e93 10.1371/journal.pbio.0030093 15757364 PMC1064851

[B33] Ludwig MZ Bergman C Patel NH Kreitman M Evidence for stabilizing selection in a eukaryotic enhancer element Nature 2000 403 6769 564 567 10.1038/35000615 10676967

[B34] Wong WSW Nielsen R Detecting selection in noncoding regions of nucleotide sequences Genetics 2004 167 2 949 958 10.1534/genetics.102.010959 15238543 PMC1470900

[B35] Pollard DA Moses AM Iyer VN Eisen MB Detecting the limits of regulatory element conservation and divergence estimation using pairwise and multiple alignments BMC Bioinformatics 2006 7 376 10.1186/1471-2105-7-376 16904011 PMC1613255

[B36] Stanojeviæ D Hoey T Levine M Sequence-specific DNA-binding activities of the gap proteins encoded by hunchback and Krüppel in Drosophila Nature 1989 341 6240 331 335 10.1038/341331a0 2507923

[B37] Kim J Macro-evolution of the hairy enhancer in Drosophila species The Journal of Experimental Zoology 2001 291 2 175 185 10.1002/jez.1067 11479916

[B38] Lavery R Recognizing DNA Quarterly Reviews of Biophysics 2005 38 04 339 344 10.1017/S0033583505004105 16515738

[B39] Sikder D Kodadek T Genomic studies of transcription factor-DNA interactions Current Opinion in Chemical Biology 2005 9 1 38 45 10.1016/j.cbpa.2004.12.008 15701451

[B40] Bulyk ML Protein binding microarrays for the characterization of DNA-protein interactions Advances in Biochemical Engineering/Biotechnology 2007 104 65 85 full_text 17290819 10.1007/10_025 PMC2727742

[B41] Alleyne TM Peña-Castillo L Badis G Talukder S Berger MF Gehrke AR Philippakis AA Bulyk ML Morris QD Hughes TR Predicting the binding preference of transcription factors to individual DNA k-mers Bioinformatics 2009 25 8 1012 1018 10.1093/bioinformatics/btn645 19088121 PMC2666811

[B42] Thorne JL Kishino H Felsenstein J Inching toward reality: an improved likelihood model of sequence evolution Journal of Molecular Evolution 1992 34 1 3 16 10.1007/BF00163848 1556741

[B43] Hwang DG Green P Bayesian Markov chain Monte Carlo sequence analysis reveals varying neutral substitution patterns in mammalian evolution Proceedings of the National Academy of Sciences of the United States of America 2004 101 39 13994 14001 10.1073/pnas.0404142101 15292512 PMC521089

[B44] Siepel A Haussler D Phylogenetic estimation of context-dependent substitution rates by maximum likelihood Molecular Biology and Evolution 2004 21 3 468 488 10.1093/molbev/msh039 14660683

[B45] Berman BP Nibu Y Pfeiffer BD Tomancak P Celniker SE Levine M Exploiting transcription factor binding site clustering to identify cis-regulatory modules involved in pattern formation in the Drosophila genome Proceedings of the National Academy of Sciences of the United States of America 2002 99 2 757 62 10.1073/pnas.231608898 11805330 PMC117378

[B46] Karolchik D Kuhn RM Baertsch R Barber GP Clawson H Diekhans M The UCSC Genome Browser Database: 2008 update Nucl Acids Res 2008 36 suppl_1 D773 779 18086701 10.1093/nar/gkm966 PMC2238835

[B47] Brudno M Do CB Cooper GM Kim MF Davydov E Green ED LAGAN and Multi-LAGAN: efficient tools for large-scale multiple alignment of genomic DNA Genome Research 2003 13 4 721 731 10.1101/gr.926603 12654723 PMC430158

[B48] Clark AG Eisen MB Smith DR Bergman CM Oliver B Markow TA Evolution of genes and genomes on the Drosophila phylogeny Nature 2007 450 7167 203 218 10.1038/nature06341 17994087

[B49] Yang Z PAML 4: phylogenetic analysis by maximum likelihood Molecular Biology and Evolution 2007 24 8 1586 1591 10.1093/molbev/msm088 17483113

[B50] Durbin R Eddy SR Krogh Mitchison G Biological sequence analysis 1998 Cambridge University Press

[B51] Felsenstein J Evolutionary trees from DNA sequences: a maximum likelihood approach Journal of Molecular Evolution 1981 17 6 368 76 10.1007/BF01734359 7288891

[B52] Bergman CM Carlson JW Celniker SE Drosophila DNase I footprint database: a systematic genome annotation of transcription factor binding sites in the fruitfly, Drosophila melanogaster Bioinformatics 2005 21 8 1747 1749 10.1093/bioinformatics/bti173 15572468

