# Supplementary material for: Statistical tests for natural selection on regulatory regions based on the strength of transcription factor binding sites
Source: BMC Evol Biol. 2009 Dec 9;9:286. doi: 10.1186/1471-2148-9-286 (PMC2800119; doi:10.1186/1471-2148-9-286)
Supplement: Additional file 1 — eve stripe 2 enhancer binding sites. Alignments of binding sites in the eve stripe 2 enhancer [file 1471-2148-9-286-S1.PDF]

# Alignments of binding sites in the *eve* stripe 2 enhancer

| BC-5 | BC-4 | BC-3 | BC-2 | BC-1 | |
|---|---|---|---|---|---|
| TTAATCCG | AAGATTAT | | AGGATTAG | GGGATTAA | pseudoobscura |
| TTAATCC**C** | CAGATTAT | TCCTTGGC | CGGATTAG | -GGATTAC | ananassae |
| TTAATCCG | CAGATTAT | **GC**A**C**TCG**G** | GGGATTAG | **C**GGATTAG | yakuba |
| TTAATCCG | CAGATTAT | AT**G**TTCGC | GGGATTAG | **C**GGATTAG | erecta |
| TTAATCCG | **C**AGATTAT | **C**TA**A**TCGC | GGGATTAG | GGGATTAG | simulans |
| TTAATCCG | **G**AGATTAT | **C**TA**A**TCGC | GGGATTAG | GGGATTAG | sechellia |
| TTAATCCG | **G**AGATTAT | ATA**A**TCGC | GGGATTAG | GGGATTAG | melanogaster |
| | | | | | |
| 01234567 | 76543210 | 01234567 | 76543210 | 76543210 | |

| KR-6 | KR-5 | KR-4 | KR-3 | KR-2 | KR-1 |
|---|---|---|---|---|---|
| TAACCCAATA | AATCCGTTTG | GACCAAGGGTTG | CGAAGGGATT | AATCGGGTTA | TAACCCCTTG |
| TAACCCAATA | AATCC**C**TTT**T** | AAC**G**--GGGTTG | CGAA-GGATT | **G**ACCGGGTTA | **C**A**G**CCCTTTT |
| TAACCCAATA | AATCCGTTTG | AACC--GGGTTG | CGAA**C**GGATT | AAC**T**GGGTTA | TAACCCGTTT |
| TAACCCAATA | AATCCGTTTG | AACC--GGGTTG | CGAA**C**GGATT | AAC**T**GGGTTA | TAACCCGTTT |
| TAACCCAATA | AATCCGTTTG | AACC--GGGTTG | CGAAGGGATT | AAC**T**GGGTTA | TAACCCGTTT |
| TAACCCAATA | AATCCGTTTG | AACC--GGGTTG | CGAAGGGATT | AAC**T**GGGTTA | TAACCCGTTT |
| TAACCCAATA | AATCCGTTTG | **G**ACC--GGGTTG | CGAAGGGATT | AAC**T**GGGTTA | TAACCCGTTT |
| | | | | | |
| 9876543210 | 9876543210 | 0123  456789 | 0123456789 | 0123456789 | 9876543210 |
